# Melanoma-initiating cells exploit M2 macrophage TGFβ and arginase pathway for survival and proliferation

**DOI:** 10.18632/oncotarget.2482

**Published:** 2014-09-16

**Authors:** Muly Tham, Kar Wai Tan, Jo Keeble, Xiaojie Wang, Sandra Hubert, Luke Barron, Nguan Soon Tan, Masashi Kato, Armelle Prevost-Blondel, Veronique Angeli, Jean-Pierre Abastado

**Affiliations:** ^1^ Singapore Immunology Network, BMSI, A-STAR, Singapore; ^2^ Laboratory of Parasitic Diseases, National Institute of Allergy and Infectious Diseases, National Institutes of Health, Bethesda, MD, USA; ^3^ School of Biological Sciences, Nanyang Technological University, Singapore; ^4^ Department of Occupational and Environmental Health, Nagoya University Graduate School of Medicine, Japan; ^5^ Institut Cochin, Université Paris Descartes, CNRS UMR, Paris, France; ^6^ Department of Microbiology, Yong Loo Lin School of Medicine, National University of Singapore, Singapore; ^7^ Department of Clinical Research, Singapore General Hospital, Singapore; ^8^ Institut de Recherche Internationales Servier, 50 rue Carnot, Suresnes cedex, France

**Keywords:** Arginase, Macrophages, TGFβ, Tumor-initiating cell

## Abstract

M2 macrophages promote tumor growth and metastasis, but their interactions with specific tumor cell populations are poorly characterized. Using a mouse model of spontaneous melanoma, we showed that CD34^−^ but not CD34^+^ tumor-initiating cells (TICs) depend on M2 macrophages for survival and proliferation. Tumor-associated macrophages (TAMs) and macrophage-conditioned media protected CD34^−^ TICs from chemotherapy *in vitro*. *In vivo*, while inhibition of CD115 suppressed the macrophage-dependent CD34^−^ TIC population, chemotherapy accelerated its development. The ability of TICs to respond to TAMs was acquired during melanoma progression and immediately preceded a surge in metastatic outgrowth. TAM-derived transforming growth factor-β (TGFβ) and polyamines produced via the Arginase pathway were critical for stimulation of TICs and synergized to promote their growth.

## INTRODUCTION

Tumor initiating cells (TICs) are stem-like cells with the ability to initiate tumor growth and contribute to common properties of tumors, including resistance to chemotherapy, recurrence following initial remission, cellular heterogeneity, and metastasis. Thus, a better understanding of the biology of TIC is essential to advance the development of novel therapies. In the past decade, the intimate relationship between the immune system and tumor progression has been recognized. Several studies have revealed possible interactions between TICs and immune cell populations; for example TICs can activate immune-suppressive T regulatory cells [1, 2], while CD8^+^ T cells support the development of TICs in breast cancer [3].

Macrophages can be broadly divided into two subtypes, M1 and M2 [4]. M1 cells are involved in pathogen clearance and pro-inflammatory responses while M2 cells are anti-inflammatory, promote tissue remodeling and are associated with tumor progression [5]. Recent studies in mice transplanted with human cancer cell lines, provided some evidence that TIC-mediated tumorigenesis and chemoresistance are supported by tumor-associated macrophages (TAMs) [6-8]. While transplanted models are a useful tool to study TICs in human cell lines, there are limitations in their ability to mimic natural tumor progression and the complexity of the tumor niche. Transplanted tumors generally develop rapidly, within days or weeks, compared to the much slower progression, months to years, observed in humans. Consequently, the composition of tumor cells, stromal cells and immune cells within transplanted tumors and their interactions may differ significantly from spontaneous tumors that develop over a prolonged period of time.

Immune-competent RETAAD mice express the human *RET* oncogene in melanocytes, resulting in uveal melanomas starting from two to three weeks of age, followed by distant metastasis. Although disseminated tumor cells are detected throughout the body from three weeks of age, metastatic outgrowth is delayed by the immune system. The median time for onset of metastatic growth are 66 days (facial), 80 days (neck/trunk), 242 days (reproductive tract) 263 days (mediastinum) and 347 days (lungs) respectively [9]. Thus the TIC-immune interaction might be a critical determinant of tumor cell quiescence, proliferation or metastasis. These mice provide a unique opportunity to study the spontaneous initiation and progression of cancer that recapitulate many features of human disease.

In this study we employed this clinically-relevant RETAAD model to analyze the interactions between TICs and TAMs in spontaneous melanomas. TICs can be detected by culturing dissociated tumor cells in a defined serum-free medium to form free-floating colonies similar to the neurosphere assay [10]. The tumor sphere culture has been used to enrich for TICs in many types of cancer including melanoma [11, 12], colon cancer [13], lung cancer [14] and breast cancer [15]. These authors have showed that cells from tumor spheres not only have more stem cell properties but also initiate tumors *in vivo* more efficiently than their adherent counterparts. Each colony, referred to as a melanosphere in the case of melanoma, is assumed to originate from a single TIC. Using this assay we seek to determine whether TAMs interact with TICs and how do these interactions affect tumor progression and response to chemotherapy? What are the underlying molecular mechanisms and pathways? And can we identify any opportunity for novel therapeutic interventions that target TIC-TAM interactions?

## RESULTS

### RETAAD tumors contain multiple tumorigenic cell subsets

**Figure 1:**
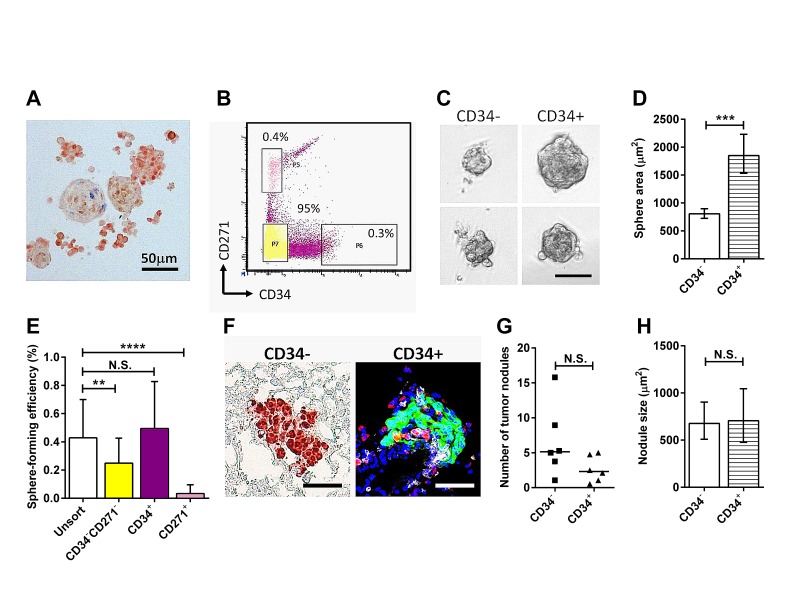
Characteristics of melanoma sub-populations in the RETAAD tumor Statistical analysis: ***P < 0.001, **** P < 0.0001, N.S. not significant. (A) Melanospheres labeled for S100B (red) and Ki67 (blue). (B) Flow cytometry with CD34 and CD271 antibodies did not reveal distinct populations. Three populations were selected as shown. (C) Brightfield images of spheres cultured from CD34^−^CD271^−^ and CD34^+^ cells. Scale bar equals 50μm. (D) Graph comparing the size of CD34^−^ and CD34+ spheres. Bars represent geometric mean ± 95% CI, two-tailed Mann-Whitney test. (E) Graph showing sphere-forming efficiency (percentage of seeded cells that formed spheres) from selected tumor cell populations indicated in B. Bars represent mean ± SD, 1-way ANOVA. (F) CD34^−^ and CD34^+^ spheres initiate tumors in the lungs of Rag1 mice. Left panel, IHC staining to illustrate lung morphology and CD34^−^ sphere initiated tumor labeled for S100B (red). Right panel, fluorescence staining to show localization of CD34^+^ sphere initiated tumor labeled for S100B (green), CD45^+^ cells (white), CD68^+^ cells (red) and nuclei (blue). Scale bars equal 50μm. (G) Comparison of number of tumor nodule presented in F. Twenty lung sections were sampled from each mouse. Each point represent one mouse, Wilcoxon matched-pairs signed rank test. (H) Comparison of size of tumor nodules presented in F. Bars represent geometric mean ± 95% CI, two-tailed Mann-Whitney test.

Using the sphere forming assay we first established that cells from the primary eye tumor forms melanospheres in culture. Consistent with the expected stem cell property of sphere-forming cells, the melanospheres could be passaged at least twice in culture, while retaining expression of the melanoma antigen S100B (Figure 1A). The phenotype of murine TICs is incompletely defined. Following the work of Held et al. [16], we studied the tumor cell populations expressing the melanocytic stem/progenitor cell marker CD34, and CD271, a neural crest stem cell marker. Unlike the previous study, RETAAD tumor cells could not be clearly separated using these two markers (Figure 1B). Therefore we isolated cells at the extreme end of the expression spectrum for each marker and compared their sphere-forming ability. Ninety-five percent of the RETAAD tumor cells were CD34^−^ CD271^−^ (hereafter denoted as CD34^−^), while the CD34 and CD271 single positive populations accounted for less than one percent. We did not observe a distinct double positive population. The CD34^+^ population formed significantly larger spheres and with higher efficiency than the CD34^−^ cells (Figure 1C to E). The CD271^+^ population formed very few spheres (Figure 1E), and therefore was not analyzed further. To determine whether the CD34^−^ and CD34^+^ sphere-forming cells could initiate tumors *in vivo*, we inoculated Rag1 mice with 100 spheres from either CD34^−^ or CD34^+^ tumor cells via the retro-orbital sinus, delivering them directly to the lungs. Despite differences in *in vitro* sphere-forming assays, both types of spheres initiated tumors *in vivo* (Figure 1F) with similar efficiency (Figure 1G) and growth rate (Figure 1H).

### The CD34^−^ population includes TICs that respond to immune cell stimulation

**Figure 2:**
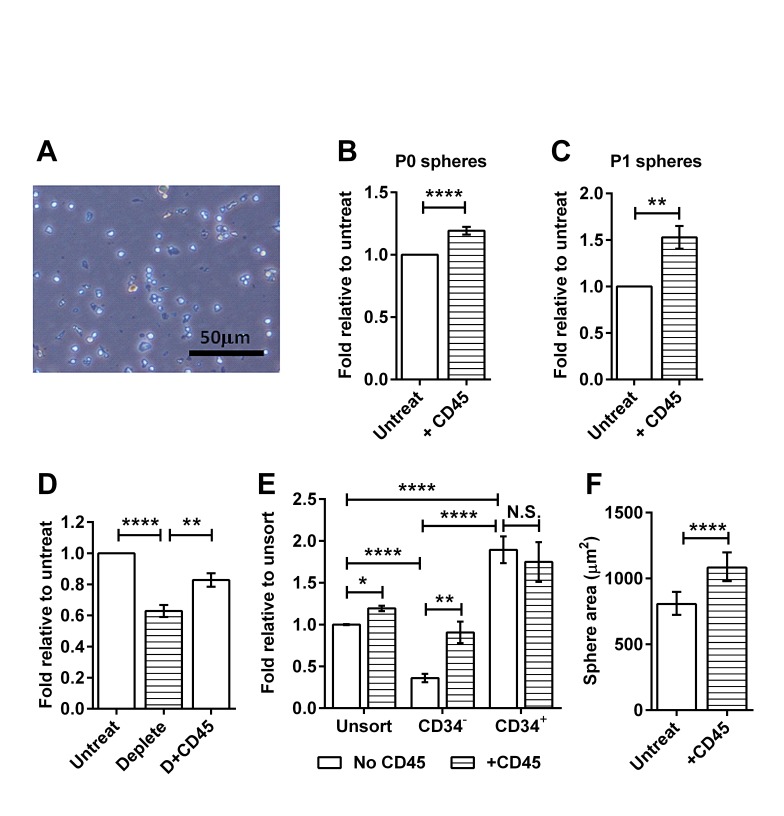
CD45^+^ immune cells stimulate melanosphere formation For panels B to E, data are expressed as fold change in sphere-forming efficiency relative to untreated or unsorted cells as indicated on the y-axis. Bars represent mean ± SE, * P < 0.05, **P < 0.01, **** P < 0.0001, N.S. not significant. (A) Brightfield image of CD45^+^ cells cultured alone in stem cell medium for 5 days. (B) Passage 0: Freshly isolated unsorted tumor cells were cultured with or without CD45^+^ immune cells isolated from tumors, two-tailed Mann-Whitney test. (C) Passage 1: Untreated P0 spheres were dissociated and recultured with or without CD45^+^ immune cells isolated from tumors, two-tailed Mann-Whitney test. (D) Graph comparing sphere-forming efficiency of untreated tumor cells, tumor cell preparations with CD45^+^ cells removed (deplete) and CD45-depleted tumor cell preparations with CD45^+^ cells added back (D+CD45), 1-way ANOVA. (E) Graph comparing sphere-forming efficiency from sorted CD34^−^ and CD34^+^ TICs cultured with or without CD45^+^ cells, 2-way ANOVA. (F) Effect of CD45^+^ cells on the size of CD34^−^ spheres. Bars represent geometric mean ± 95% CI, two-tailed Mann-Whitney test.

Based on the emerging evidence of interactions between immune cells and TICs, we asked whether tumor-associated immune cells affect the ability of the TICs to form spheres in culture. We purified CD45^+^ immune cells from tumors and added them to TICs in sphere cultures at a ratio of 1:50, corresponding to their relative abundance in RETAAD tumors *in vivo* [17]. CD45^+^ cells cultured alone in melanosphere medium did not form spheres (Figure 2A). Adding CD45^+^ cells to unsorted tumor cells led to a slight increase in efficiency of sphere formation (P0 spheres) (Figure 2B). Similarly, dissociated cells from untreated P0 spheres continued to increase their sphere-formation efficiency in response to CD45^+^ cells (Figure 2C), implying that the immune-responsive TICs were maintained during passage. We then determined whether immune cells were involved in the normal maintenance of the TICs by depleting CD45^+^ cells from dissociated tumor cell preparations. The absence of CD45^+^ cells led to a significant reduction in sphere formation efficiency, which was rescued by re-addition of purified CD45^+^ cells (Figure 2D). As sphere formation was reduced, but not abrogated by the loss of CD45^+^ cells, we conclude that a proportion of the TICs in RETAAD tumors is dependent on immune cells for survival. To identify this population we compared the effect of CD45^+^ cells on the ability of CD34^−^ and CD34^+^ TIC sub-populations to form spheres *in vitro*, and found that only the CD34^−^ TICs responded to the addition of CD45^+^ cells (Figure 2E). In addition to increasing the efficiency of CD34^−^ TIC sphere formation, CD45^+^ cells also significantly increased sphere size (Figure 2F). Thus CD45^+^ cells promote both melanosphere initiation and proliferation.

### TAMs with M2-like properties stimulate melanosphere formation by CD34^−^ TICs

**Figure 3:**
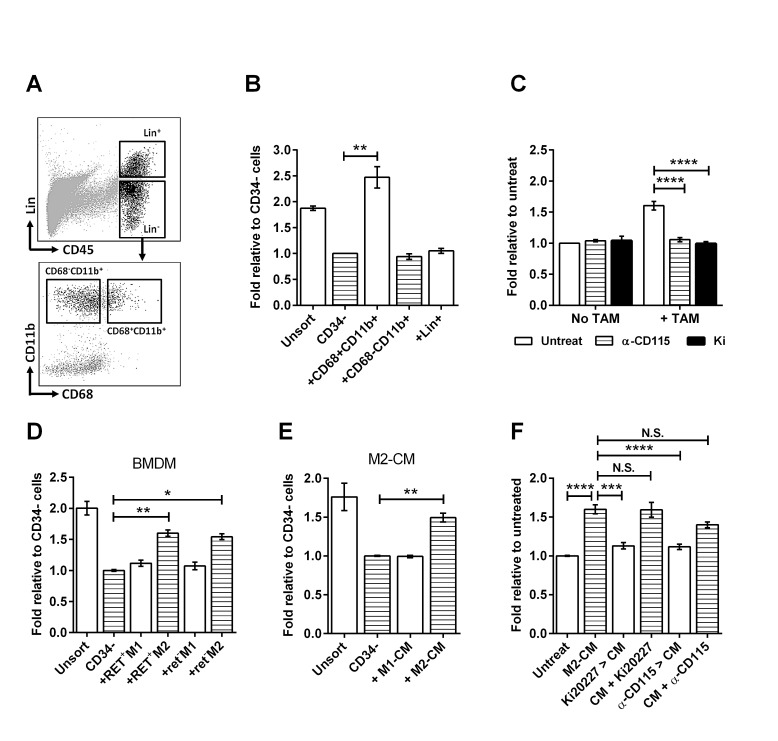
TAMs stimulate melanosphere formation Data for all bar graphs are expressed as fold change in sphere-forming efficiency relative to untreated CD34^−^ TICs. Bars represent mean ± SE, * P < 0.05, **P < 0.01, ***P < 0.001, **** P < 0.0001, N.S. not significant, 1-way ANOVA for B and D to F, 2-way ANOVA for C. (A) Flow cytometry sorting of immune cell populations. The Lin^+^ gate contain T, B and NK cells. Lin^−^ cells are separated into CD11b^+^CD68^−^ myeloid cells and CD11b^+^CD68^+^ TAMs. (B) Effect of immune cell populations on sphere formation: CD34^−^ TICs were cultured with the immune cell populations sorted in A. (C) Effect of TAM inhibition on sphere formation: CD34^−^ TICs were cultured with or without TAMs. Two CSF1R inhibitors Ki20227 (Ki; 100nM) and CD115-neutralizing antibody (α-CD115; 1μg/ml) were added for the duration of the culture. (D) Effect of BMDM on sphere formation: BMDM from tumor-bearing mice (RET^+^) and non-tumor bearing littermates (ret^−^) were polarized to M1 or M2 phenotype and co-cultured with CD34^−^ TICs. (E) Effect of conditioned medium (CM) on sphere formation: CM generated from M1- or M2-polarized BMDM were added to CD34^−^ TICs at 1/10^th^ of stem cell media volume. (F) Effect of CSF1R inhibitors on the stimulatory effect of M2-BMDM CM (M2-CM): Ki20227 (100nM) and anti-CD115 (1μg/ml) were either added to the BMDM prior to CM generation (Ki20227 > CM, α-CD115 > CM) or added to the CM during sphere culture (CM+Ki20227, CM+α-CD115).

Amongst CD45^+^ immune cells, macrophages are known to promote tumor cell proliferation and migration [18]. To determine whether macrophages are involved in stimulating sphere formation by CD34^−^ TICs, we isolated CD11b^+^CD68^+^ TAMs (Supplemental Figure S1C for morphology) from RETAAD tumors using flow cytometry (Figure 3A) and co-cultured them with CD34^−^ TICs in the sphere-forming assay. TAMs significantly increased sphere formation by CD34^−^ TICs (more than two folds), while CD11b^+^CD68^−^ myeloid cells had no effect (Figure 3B). T, B and NK cells within the Lin^+^ gate also have no stimulatory effect whether as a mixed population (Figure 3B) or as separate populations (Supplemental Figure S1A and B).

To confirm the role of TAMs in *in vitro* sphere formation by TICs we next inhibited the colony stimulatory factor-1 receptor (CSF-1R or CD115), which is required for macrophage survival and polarization [19, 20]. Using two CD115 inhibitors, the tyrosine kinase inhibitor Ki20227 and a neutralizing antibody, we showed that blocking CD115 *in vitro* abolishes the stimulatory effect of TAMs on sphere formation by CD34^−^ TICs, but does not affect sphere formation in the absence of TAMs (Figure 3C). To exclude the possibility that CD115 may be present on the tumor cells and contribute to the observed effects, we pre-treated the tumor cells with anti-CD115 antibody before stimulating them with TAMs. This did not inhibit the stimulatory effect of TAMs (Supplemental Figure S1D). Conversely, pre-treatment of TAMs with anti-CD115 antibody before adding them to the tumor cells completely abrogated their stimulatory effect (Supplemental Figure S1E). In addition, qPCR confirmed that the CD34^−^ tumor cells express negligible levels of CD115 compared to the TAMs (Supplemental Figure S1F), and CD115 immunolabeling within tumor sections was accordingly restricted to immune cells (Supplemental Figure S1G). Together these data demonstrate that CD115 inhibition prevents TAMs from stimulating sphere formation by the CD34^−^ TIC population within RETAAD primary melanomas.

Two recent publications has shown that cancer-associated fibroblasts (CAF) can promote stemness in prostate cancer cells [21] and that CAF can synergize with M2 macrophages to promote tumor invasiveness [22]. Since the CD34^−^ tumor population in our study will also include the fibroblasts and endothelial cells it is possible that the observed sphere stimulatory effects of TAMs may involve interactions with CAFs. To exclude this possibility we removed the CD45^−^ CAF based on Platelet Derived Growth Factor Receptor (PDGFR)α expression [23] and the CD45^−^ endothelial cells based on CD31 expression (Supplemental Figure S2A). Removal of these stromal cells did not alter the melanosphere formation efficiency (Supplemental Figure S2B) or affected the stimulatory effect of the TAMs on sphere formation and sphere proliferation (Supplemental Figure S2C and D). Since these stromal cells do not contribute to the ability of TAMs to stimulate sphere formation by the CD34^−^ TICs we did not eliminate them from subsequent experiments.

To gain insight into the mechanisms underlying TIC stimulation by macrophages, we analyzed the cytokine profiles of TAMs. Similar to other models [24], TAMs isolated from RETAAD tumors exhibited a gene expression pattern consistent with M2 macrophages including high expression of transforming growth factor-β (*Tgfb1*)*,* arginase 1 (*Arg1*) and *Fizz1* (Supplemental Figure S3A), with lower levels of M1-related genes such as nitric oxide synthase 2 (*Nos2*), interleukin-12 (*Il12*) and interleukin-1β (*Il1b*) (Supplemental Figure S3B). These TAMs also express the M2 macrophage marker CD206 (Supplemental Figure S3C). Thus we hypothesized that M2-like macrophages could stimulate melanosphere formation. To test this hypothesis, we polarized bone marrow-derived macrophages (BMDM) towards either the M1- or M2-phenotype using LPS and IFNγ, or IL-4 respectively. M2-BMDM from both tumor-bearing (RET^+^) mice and non-tumor bearing (ret^−^) littermates stimulated sphere formation, while the M1 macrophages had minimal activity (Figure 3D). Furthermore, conditioned medium generated from M2-BMDM (M2-CM) stimulated sphere formation while M1-conditioned medium (M1-CM) did not (Figure 3E). Pre-treatment of M2-BMDM with Ki20227 or anti-CD115 antibody before generating CM removed the stimulatory effect of CM, whereas direct addition of these inhibitors to the CM during sphere culture had minimal effect (Figure 3F). This shows that activated M2-like macrophages derived from either tumor-bearing or wild-type mice stimulate sphere formation by CD34^−^ TICs.

### The onset of CD34^−^ TIC responsiveness to TAM stimulation correlates with disease progression *in vivo*

**Figure 4:**
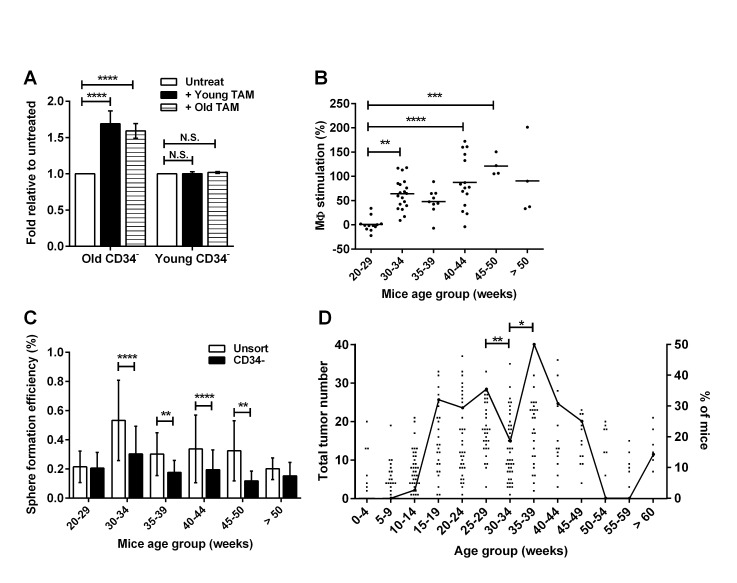
Acquisition of macrophage responsiveness by TICs coincides with tumor progression Statistical analysis: * P < 0.05, **P < 0.01, ***P < 0.001, **** P < 0.0001, N.S. not significant. (A) CD34^−^ TICs from old (>30 week) or young (<30 week) mice were cultured with TAMs derived from old or young mice. Data are expressed as fold change in sphere formation efficiency relative to untreated CD34^−^ TICs. Bars represent mean ± SE, 2-way ANOVA. (B) Percentage of macrophage stimulation of sphere formation from CD34^−^ cells from mice of different age groups, reflecting stage of disease. TAMs were donor-matched to the CD34^−^ TICs. Each point represents one mouse, 1-way ANOVA. (C) Graph comparing sphere-forming efficiency of unsorted cells and CD34^−^ TICs from mice of different age groups. Bars represent mean ± SD, 2-way ANOVA. (D) Graph showing the number of macroscopic tumors detected in mice of different ages on the left axis. Each point represents one mouse (total 285 mice). Black line is the percentage of mice (right axis) that have more than 21 tumors (80th percentile). One-way ANOVA.

Having observed the potent effects of TAMs on RETAAD TICs *in vitro* it seemed plausible that TAM-mediated TIC stimulation might play a role in melanoma progression. We first compared the responses of TICs to TAM stimulation using cells isolated from mice at various stages of disease progression. Interestingly, the CD34^−^ TICs isolated from young mice (< 30 weeks) with less advanced disease formed spheres with equal efficiency in the presence or absence of TAMs, while TICs from mice over 30 weeks of age was strongly stimulated by TAMs (Figure 4A). This phenomenon was driven by the TIC population rather than the TAMs, as “young” TICs similarly failed to respond to “old” TAMs isolated from mice with advanced disease, while TICs from older mice responded to TAMs from mice with early or late disease (Figure 4A). As shown in Figure 4B, CD34^−^ TIC responsiveness to TAMs began at 30 weeks of mouse age, peaked in mice aged between 45 and 50 weeks and then slightly decreased in mice above 50 weeks of age. Importantly, the total number of CD34^−^ TICs did not change significantly with age across the analyzed period (20 to 50 weeks) but rather a subpopulation of these TICs became responsive to TAMs after 30 weeks (Figure 4C). In order to understand the significance of this change *in vivo* we compared the timeline of *in vitro* TIC responsiveness to TAM stimulation retrospectively with necropsy data of tumor burden in mice aged 1-69 weeks. The acquisition of TAM-responsiveness from 30 weeks of mouse age immediately preceded a sharp increase in the percentage of mice having a large number of tumors (Figure 4D): 50% of the mice analyzed between 35 and 39 weeks of age had more than 21 macroscopic metastases (80^th^ percentile for tumor burden). The percentage of mice with more than 21 macroscopic metastases had a biphasic pattern (Figure 4D), implying the presence of two sub-populations of mice with early and late tumor development. Taken together, these results suggest that late on during disease progression (after 30 weeks of age) a sub-population of CD34^−^ TICs either arises or acquires the ability to respond to TAM stimulation, and so may contribute to the sudden outgrowth of a proportion of metastases at around 30 weeks of age, while those tumors developing before this time are likely originating from TICs that are independent of TAMs for survival and growth.

### TAMs support CD34^−^ TIC survival *in vivo*

**Figure 5:**
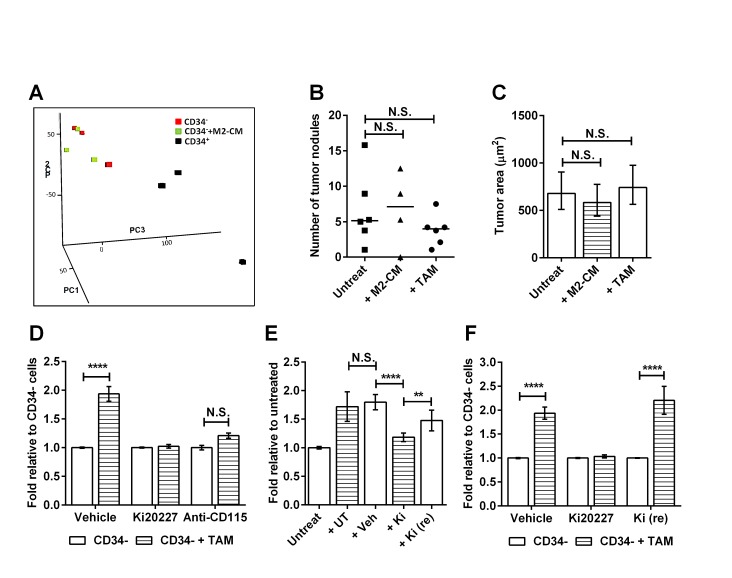
Macrophages support TIC survival *in vivo* Data for D to F are expressed as fold change in sphere-forming efficiency relative to untreated CD34^−^ TICs. Statistical analysis: **P < 0.01, **** P < 0.0001, N.S. not significant. (A) Principle Component Analysis (PCA) mapped scatter plot: The global gene expression profiles of untreated (red) or M2-CM treated CD34^−^ spheres (green) and CD34^+^ spheres (black) were analyzed by PCA. Graph represents the first three principal components of the microarray analysis data (PC1, PC2 and PC3) in the X, Y and Z axis respectively. (B) Comparison of number of tumor nodules formed in lungs of Rag1 mice injected with CD34^−^ spheres derived from untreated, M2-CM- or TAM-stimulated cultures. Twenty lung sections were sampled from each mouse. Each point represent one mouse, Wilcoxon matched-pairs signed rank test. (C) Comparison of size of the tumor nodules presented in B. Bars represent geometric mean ± 95% CI, 1-way ANOVA. (D) Effect of *in vivo* CD115 inhibition on sphere formation *in vitro*: CD34^−^ TICs from mice treated with vehicle, Ki20227 or anti-CD115 for 10 days were cultured with or without TAMs from untreated mice. Bars represent mean ± SE, 2-way ANOVA. (E) Effect of *in vivo* CD115 inhibition on TAM activity *in vitro*: CD34^−^ TICs from untreated mice were cultured with TAMs from untreated (+UT) mice, or mice treated with vehicle (+Veh), Ki20227 for 10 days (+Ki) or Ki20227 for 10 days followed by 10 days recovery [+Ki (re)] mice. Bars represent mean ± SE, 1-way ANOVA. (F) Recovery of macrophage-responsive TICs: CD34^−^ TICs from vehicle-, Ki20227- or Ki20227 recovery- treated mice were cultured with or without TAMs from untreated mice. Bars represent mean ± SE, 2-way ANOVA.

Our data show that TAMs can support CD34^−^ TIC survival and proliferation *in vitro* and that *in vivo* this may translate into increased tumor burden in older RETAAD mice. Therefore we asked whether the TAMs were mediating their effect on TICs by inducing changes in gene expression. Microarray comparison of TAM-stimulated and non-stimulated CD34^−^ melanospheres revealed comparable patterns of expression, whereas the CD34^+^ spheres had a distinct transcriptome, as shown by principal component analysis (Figure 5A). In particular TAM stimulation did not increase the expression of CD34 in the CD34^−^ spheres (Supplementary Figure S4), indicating that the TAM-stimulated CD34^−^ spheres are not becoming CD34^+^ spheres. When the same number of TAM-stimulated and non-stimulated CD34^−^ spheres were transplanted *in vivo* they initiated tumors in the lungs with similar efficiency and of similar size (Figure 5B and C). This indicated that TAMs increase CD34^−^ TIC survival (sphere formation) without altering their tumorigenic potential. To determine whether CD34^−^ TICs depend on macrophages for survival *in vivo*, we treated mice with Ki20227 or anti-CD115 antibody for 10 days. CD34^−^ TICs were isolated from treated mice and cultured with TAMs from untreated animals, but CD115 inhibition had rendered them unable to respond to TAM stimulation of sphere formation (Figure 5D). To understand how CD115 inhibition *in vivo* abolished TIC responsiveness to TAMs *in vitro*, we isolated TAMs from inhibitor-treated mice and asked whether they could stimulate TICs from untreated mice. Ki20227 treatment did not reduce the number of CD68^+^TAMs, (Supplemental Figure S5A) but significantly inhibited their sphere-stimulatory activity (Figure 5E). In contrast, treatment with anti-CD115 antibody reduced the number of TAMs by 50% (Supplemental Figure S5B), but did not affect the TIC-stimulatory activity of the residual TAMs (Supplemental Figure S5C). Taken together, these data demonstrate that a sub-population of CD34^−^ TICs depends on TAMs for survival *in vivo*, such that inhibition of TAM activity or reduction of TAM numbers causes the functional disappearance of this tumor cell population. This effect is completely reversible, as within 10 days of terminating Ki20227 treatment both TAM activity (Figure 5E) and the TAM-dependent TIC population had recovered (Figure 5F).

### Chemotherapy accelerates the emergence of macrophage-responsive TICs

**Figure 6:**
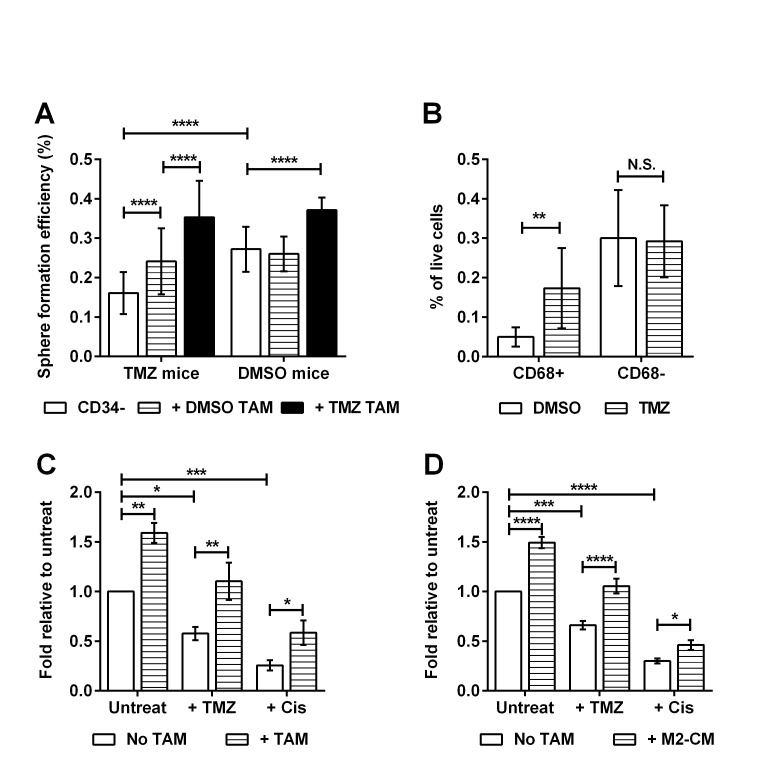
Chemotherapy promotes the emergence of macrophage-responsive TICs Data for C & D are expressed as fold change in sphere-forming efficiency relative to untreated CD34^−^ TICs. Statistical analysis: * P < 0.05, ** P<0.01, *** P<0.001, **** P < 0.0001, N.S. not significant, 2-way ANOVA. (A) Effect of Temozolomide (TMZ) on the emergence of macrophage-responsive TICs: Tumor cells from young mice (age 23-28 weeks) treated with TMZ or DMSO were stimulated with TAMs derived from TMZ or DMSO treated mice. Bars represent mean ± SD. (B) Graph comparing percentage of TAM population derived from young mice treated with TMZ or DMSO. Bars represent mean ± SD. (C) Effect of TAMs on chemotherapy-treated TICs: CD34^−^ TICs from old mice were cultured with TMZ (200μg/ml) or cisplatin (Cis, 0.5μg/ml) with or without TAMs. Bars represent mean ± SE. (D) Effect of M2-CM on chemotherapy-treated TICs: CD34^−^ TICs from old mice were cultured with TMZ or cisplatin with or without M2-CM. Bars represent mean ± SE.

Chemotherapy remains the standard of care for many types of cancer, but such treatment can select for more aggressive TIC clones [25, 26]. We therefore asked whether chemotherapy promotes the emergence of the macrophage-responsive TIC sub-population *in vivo*. We exploited the fact that TICs purified from primary tumors of RETAAD mice less than 30 weeks old did not respond to macrophage stimulation (Figure 4A) to enable us to detect any increases in TIC responsiveness as a result of chemotherapy. After three doses of temozolamide (TMZ), TICs from young mice (< 28 weeks) became responsive to stimulation of sphere-formation by TAMs derived from either control (DMSO-) or TMZ-treated mice (Figure 6A). Remarkably, TMZ also increased the stimulatory activity of the TAMs such that even normally unresponsive TICs purified from DMSO-treated mice responded to TAMs purified from TMZ-treated mice. Furthermore, chemotherapy increased the percentage of CD68^+^ TAMs within the tumor (Figure 6B). When TICs from untreated mice were exposed to TMZ or another chemotherapy drug, cisplatin *in vitro*, sphere formation was reduced by more than 50% (Figure 6C), but TAMs protected the CD34^−^ TICs from these adverse effects. M2-CM also protected CD34^−^ TICs from TMZ and cisplatin treatments (Figure 6D). These results demonstrate that chemotherapy recruits macrophages into the tumor, accelerates the development of TAM-responsive TICs *in vivo* and that TAMs also protect these TICs from chemotherapy.

### Macrophages stimulate melanosphere formation via TGFβ and the Arginase pathway

**Figure 7:**
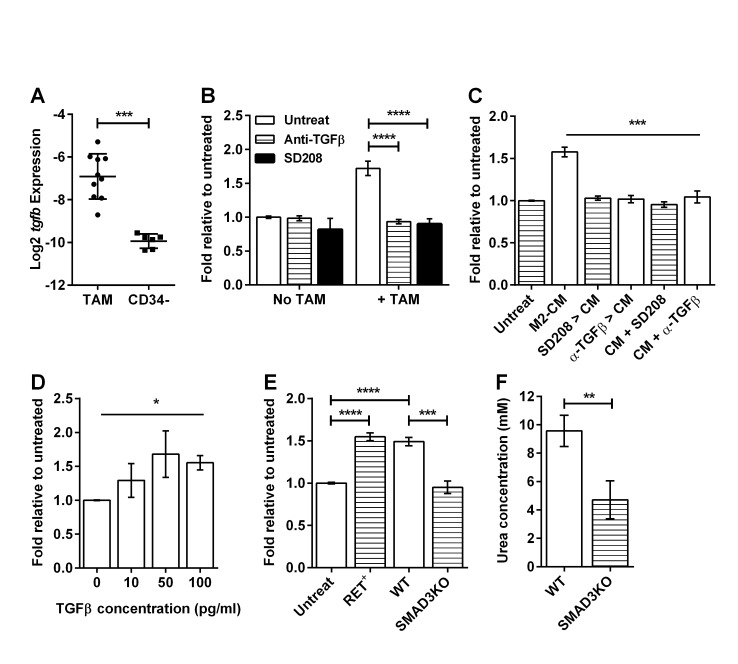
Macrophages stimulate melanosphere formation via TGFβ For panels (B) to (E), data are expressed as fold change in sphere-forming efficiency relative to untreated CD34^−^ TICs. Statistical analysis: * P<0.05, ** P<0.01, *** P<0.001, **** P < 0.0001. (A) Graph comparing the Log2 expression of the *tgfb* gene relative to GAPDH in TAMs and CD34^−^ TICs. Bars represent mean ± SD with each point representing cells from one mouse, two-tailed Mann-Whitney test. (B) Effect of TGFβ inhibitors on the stimulatory effect of TAMs: Anti-TGFβ antibody (1μg/ml) and SD208 (1μM) were added to CD34^−^ TIC cultures with or without TAMs for the duration of the experiment. Bars represent mean ± SE, 2-way ANOVA. (C) Effect of TGFβ inhibitors on the stimulatory effect of M2-CM: Anti-TGFβ and SD208 were either added to the BMDM prior to CM generation (SD208 > CM, α-TGFβ > CM) or added to the CM during sphere culture (CM+SD208, CM+α-TGFβ). Bars represent mean ± SE. Line above bars represents comparison between M2-CM and each of the treatments, 1-way ANOVA. (D) Graph showing the dose-dependent stimulatory effect of TGFβ on sphere formation. Bars represent mean ± SE. Line above bars represent analysis of linear trend, 1-way ANOVA. (E) Effect of M2-BMDM derived from tumor-bearing mice (RET^+^), wild-type mice (WT) and Smad3 knockout mice (SMAD3KO) on sphere formation. Bars represent mean ± SE, 1-way ANOVA. (F) Comparison of Arg1 activity within WT and SMAD3KO M2-BMDM reflected by the amount of urea production. Bars represent mean ± SD, two-tailed Mann-Whitney test.

Our data indicate that activated M2-like macrophages stimulate sphere formation from CD34^−^ TICs via secreted factor(s) (Figure 3E and F). TAMs from RETAAD tumors contain high levels of *Tgfb* mRNA compared to tumor cells (Figure 7A). As TGFβ is well known to regulate tumor growth and metastasis [27], and supports the maintenance of stem cells [28], we hypothesized that TAM-derived TGFβ might be involved in promoting CD34^−^ TIC survival. To test this hypothesis we inhibited TGFβ signaling in our melanosphere assays using either SD208 [29], a small molecule TGFβ receptor kinase inhibitor, or a neutralizing antibody against TGFβ. Both inhibitors abolished the sphere stimulatory activity of TAMs, without affecting sphere formation in the absence of TAMs (Figure 7B). Both inhibitors also abolished the stimulatory effect of BMDM derived M2-CM when added directly to the sphere culture (Figure 7C), showing that TGFβ is present in the M2-CM and that the TICs must be responding via the TGFβ receptor. Accordingly, stimulation of TICs with recombinant TGFβ increased melanosphere formation in a dose-dependent manner (Figure 7D). Interestingly, M2-BMDM pretreated with SD208 or anti-TGFβ antibody generate CM that no longer stimulate sphere formation (Figure 7C). Therefore, in addition to direct effects on the TICs, TGFβ also acts upon the TAMs to enable downstream production of additional soluble factors that contribute to stimulation of sphere formation by TICs. Smad3 is a transcription factor activated by TGFβ and Smad3 knockout (Smad3KO) mice have defective TGFβ signaling [30]. Accordingly, M2-BMDM and M2-CM derived from Smad3KO mice were unable to stimulate melanosphere formation by CD34^−^ TICs (Figure 7E and Supplemental Figure S6A). This confirms that signaling downstream of TGFβ within macrophages is required for production of additional stimulatory factors. Two known functions of TGFβ are auto-regulation [31] and regulation of Arginase 1 (Arg1) activity [32], leading to production of polyamines that are essential for cell growth and differentiation. We analyzed CM from wild-type (WT) and Smad3KO M2-BMDMs and found no significant difference in TGFβ concentration (Supplemental Figure S6B). However Smad3KO cells had significantly lower Arg1 activity (Figure 7F).

**Figure 8:**
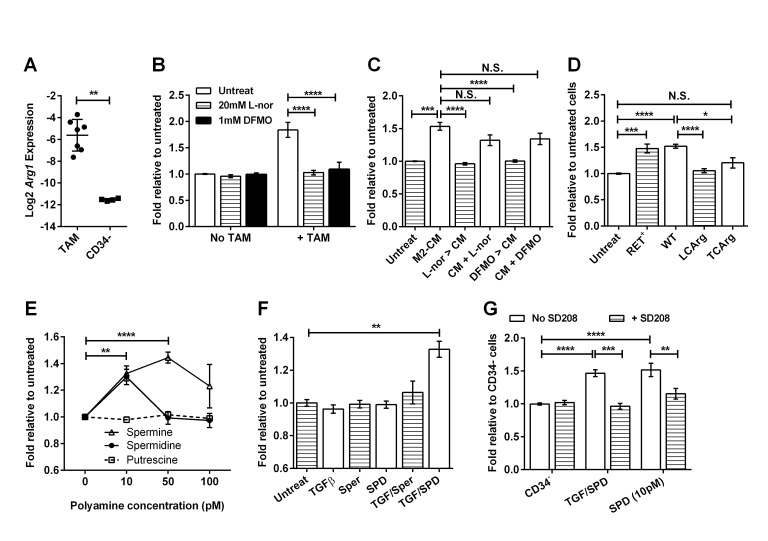
Macrophages stimulate melanosphere formation via the Arginase 1 pathway For panels (B) to (G), data are expressed as fold change in sphere-forming efficiency relative to untreated CD34^−^ TICs, bars represent mean ± SE. Statistical analysis: * P<0.05, ** P<0.01, *** P<0.001, **** P < 0.0001, N.S. not significant. (A) Graph comparing the Log2 expression of the *Arg1* gene relative to GAPDH in TAMs and CD34^−^ TICs. Bars represent mean ± SD, two tailed Mann-Whitney test. (B) Effect of arginase pathway inhibitors on the stimulatory effect of TAMs: L-norvaline (L-nor; 20mM) and DFMO (1mM) were added to CD34^−^ TIC cultures with or without TAMs for the duration of the experiment, 2-way ANOVA. (C) Effect of arginase pathway inhibitors on the stimulatory effect of M2-CM: L-nor and DFMO were either added to the BMDM prior to CM generation (L-nor > CM, DFMO > CM) or added to the CM (CM+L-nor, CM+DFMO) during sphere culture, 1-way ANOVA. (D) Effect of M2-BMDM derived from tumor bearing mice (RET^+^), *Arg1^fl/fl^* mice (WT), *Arg1^fl/fl^;LysMcre* mice (LCArg) and *Arg1^fl/fl^;Tie2cre* mice (TCArg) on sphere formation, 1-way ANOVA. (E) Effect of increasing concentrations of recombinant polyamines on sphere formation, 2-way ANOVA. (F) Synergistic effect of TGFβ and spermidine (SPD) on sphere formation: TGFβ (5pg/ml), spermine (Sper; 5pM) and SPD (5pM) were added individually or in the combinations shown, 2-way ANOVA. (G) Effect of TGFβ receptor inhibition on TGFβ- and SPD- stimulated sphere formation: CD34^−^ TICs were cultured alone, with TGFβ (5pg/ml) and SPD (5pM), or with SPD (10pM) in the presence or absence of SD208 (1μM), 2-way ANOVA.

The production of polyamines via Arginase 1 can stimulate tumor growth [33], and TAMs from RETAAD tumors expressed high levels of *Arg1* (Figure 8A). To determine whether Arg1 activity and the associated production of polyamines contributed to TAM stimulation of sphere formation from CD34^−^ TICs, we used two inhibitors of this pathway: L-norvaline blocks Arg1 activity by competing with the Arg1 substrate L-arginine, while difluoromethyl ornithine (DFMO) inhibits ornithine decarboxylase, an enzyme downstream of Arg1 that converts L-ornithine to polyamines. Both inhibitors specifically blocked the stimulatory effect of TAMs without affecting baseline sphere formation by TICs (Figure 8B). Both inhibitors also eliminated the stimulatory effect of M2-CM when BMDM were treated prior to CM generation (Figure 8C). When the inhibitors were added to untreated M2-CM during sphere culture, the stimulatory effect of M2-CM was reduced slightly. This shows that both inhibitors predominantly inhibit the macrophage Arg1 pathway, but may also act directly on the tumor cells. To avoid the non-specific activity of these inhibitors on the tumor cells, we used M2-polarized BMDM from mice with macrophage-specific deletion of *Arg1*. The *Arg1^fl/fl^; LysMcre* (LCArg) mice lack *Arg1* expression in macrophages and neutrophils, while the *Arg1^fl/fl^; Tie2cre* (TCArg) mice lack *Arg*1 in all hematopoietic lineage cells [34]. While the M2-BMDM from the *Arg1^fl/fl^* control mice stimulated TIC sphere formation normally, both the LCArg and TCArg mutant M2-BMDMs no longer stimulated sphere formation from CD34^−^ TICs (Figure 8D). Similarly, CM derived from these mutant BMDMs showed no stimulatory effect but contained normal levels of TGFβ (Supplemental Figure S6C and D). Arg1 activity in both mutant BMDMs was severely reduced (Supplemental Figure S6E), confirming that Arg1 activity is essential for macrophages to stimulate sphere formation. Moreover, addition of exogenous polyamines to TICs, specifically spermine and spermidine, recapitulated the stimulatory effect of TAMs and M2-CM on sphere formation (Figure 8E). The involvement of Arg1 is also confirmed when we looked at the effect of TMZ treatment *in vivo* and found that TMZ significantly increased the expression of Arg1 in TAMs (Supplementary Figure S7A) correlating with the increased stimulatory effect of the TAMs (Figure 6A). Conversely, inhibition of CD115 using Ki20227 in vivo and *in vitro*, reduced the expression and activity of Arg1 (Supplementary Figure S7B – D) correlating with reduced TAM activity (Figures 3C and 5D). The effect of anti-CD115 antibody *in vitro* is similar, but its effect *in vivo* is likely due to the reduction in macrophage numbers rather than activity (Supplemental Figure S5B and C). These treatments did not alter the expression of TGFβ (Supplementary Figure S7E – G).

Paradoxically, addition of TGFβ or polyamines alone was sufficient to stimulate sphere formation yet inhibiting either abolished the TAM and M2-CM sphere-stimulatory effects. This suggests that under normal conditions TAMs produce limited amounts of each molecule such that both are necessary to stimulate melanosphere formation. Accordingly, sub-stimulatory amounts of both TGFβ (5pg/ml) and spermidine (5pM) synergized to achieve sphere stimulation (Figure 8F). Furthermore, inhibition of TGFβ signaling by SD208 prevented both this synergy of TGFβ and spermidine, and also prevented sphere stimulation by higher dose spermidine alone (Figure 8G), indicating that spermidine signaling is dependent on an intact TGFβ pathway.

## DISCUSSION

Disease progression and resistance to treatment has been attributed to TICs in several types of cancer. Despite recognition of the intimate relationship between the immune system and tumor progression, to date, TICs have mostly been studied in immune-deficient xenograft models. In the present study, we used immune-competent mice that spontaneously develop progressive melanoma that recapitulates many important features of the human disease. We identified two distinct populations of TICs of which the CD34^−^ TIC sub-population was uniquely responsive to TAMs, which increased both TIC survival and proliferation through the TGFβ and Arginase pathways. Although the CD34^+^ population also efficiently initiated tumors *in vivo*, its relative scarcity means that its biological significance is likely to be negligible compared to the CD34^−^ TICs.

Macrophages, specifically TAMs and the related subset of M2-/alternatively-activated macrophages promote tumor cell proliferation and extravasation at the metastatic site [35]. In patients, the number of TAMs are generally correlated with poor prognosis [36]. Indeed, a recently approved chemotherapeutic agent, trabectedin, may reduce tumor growth in part by depleting TAMs [37]. Our data reveal that TAMs in the primary tumor also promote tumor initiation. Although TAMs do not alter the gene expression profile or the tumorigenic ability of TICs, they are essential for the survival of TICs as indicated by the ability of TAMs to stimulate sphere formation. This suggests that the presence of TAMs can increase the opportunities for tumor growth to be initiated *in vivo*. The ability of these TAM-responsive TICs to disappear with CSF-1R inhibition and recover rapidly upon termination of macrophage inhibition suggests that the TIC phenotype may be plastic. This is consistent with the ability of melanoma cells to switch phenotypes [38], and suggest that alterations to the tumor microenvironment might change the behavior of tumor cells, including TICs. Indeed, chemotherapy induced rapid appearance of a macrophage-responsive TIC population in young RETAAD mice with early disease, while macrophages protect this TIC population from chemotherapy by stimulating more sphere formation. TMZ treatment also increased Arginase 1 expression and the corresponding stimulatory activity of TAMs. Furthermore, chemotherapy promotes macrophage infiltration into the tumor (our data and ref.[8, 39]) and TAM density correlates with increased tumor relapse [40, 41]. Thus, our data suggest that TIC-macrophage interactions could contribute to relapse after chemotherapy.

An interesting phenomenon that we observed is that TICs from young mice (< 30wks) do not respond to TAM stimulation. There are several possible explanations for this. Firstly, tumors in young mice may be more self-sufficient. But as the disease progresses, limitations within the tumor microenvironment including nutrient and space may force tumor cells to adapt and utilize all the available cytokines, including those from TAMs within their environment to survive and propagate. Alternatively, the TAM-dependent TICs that developed after 30 weeks of age may be a new tumor cell clone that did not exist previously. More work will be required to elucidate this point.

Using our registry of 285 clinically-characterized RETAAD mice we show that the rate of spontaneous metastasis development varies with age and that metastases occur in two waves. A sharp increase in metastases after 30 weeks coincides with the acquisition of macrophage responsiveness by CD34^−^ TICs. Currently we do not know whether this second wave of metastases develop from dormant early disseminated TICs acquiring the ability to respond to growth stimuli from macrophages, or from new populations of macrophage-responsive TIC clones leaving the primary tumor. In any case, macrophage inhibition *in vivo* suppressed the macrophage-responsive TIC population. Human cancers have similar heterogeneous rates of progression [42, 43], suggesting some heterogeneity in human TICs as well, and illustrating the advantages of using clinically-relevant mouse models to dissect these phenomena.

Recent studies showed that tumor-educated macrophages can stimulate TICs [6-8]. In contrast, our data show that the M2 phenotype, i.e. the production of TGFβ and polyamines, is sufficient to stimulate TIC survival regardless of tumor exposure since macrophages from non-tumor bearing mice also stimulated TIC survival (Figure 3D). Thus, the presence of M2 macrophages in organs such as the lungs may contribute to their marked susceptibility as sites of metastasis by enhancing the survival of any disseminated TIC that arrives. We have unpublished data indicating that lung-derived macrophages, whether from healthy or tumor-bearing mice, can stimulate CD34^−^ TICs. Indeed, macrophages may even contribute to the attraction of stem cells as demonstrated by the ability of macrophages to recruit human vessel-associated stem cells for muscle repair [44].

Consistent with the adaptability of tumor cells, we found that CD34^−^ TICs use existing trophic pathways within their environment, namely TAM-derived TGFβ and polyamines, for their survival. The late (>30 weeks of age) acquisition of TGFβ/TAM-dependent survival in our model is consistent with TGFβ being a tumor suppressor during early carcinogenesis but promote tumor growth and metastasis with tumor progression [27]. TAM-derived TGFβ may also suppress the immune system [45, 46], and convert non-TICs to TICs due to its ability to induce epithelial-mesenchymal transition (EMT) [47], which in turn promotes “stemness” in non-TICs [3, 48], though more work will be required to confirm these hypotheses.

Polyamines are essential for cell growth and are frequently dysregulated in cancer [49]; with multiple polyamine inhibitors currently in clinical trials as cancer treatments [50, 51]. Besides tumor-derived polyamines, macrophages are also an important source of polyamines within the tumor microenvironment [33, 52]. The dependency on TAM-derived polyamines suggests that the TICs might lack polyamines or unable to consume them without TAM stimulation. The synergism between TGFβ and spermidine suggest that TGFβ might regulate polyamine uptake similar to its regulation of arginine uptake in vascular smooth muscle cells [53], and indicates that even in tumors with few infiltrating macrophages, low levels of macrophage-derived TGFβ and polyamines could contribute to TIC survival and metastasis.

CAFs are also an important source of TGFβ within the tumor microenvironment [54] and can stimulate cancer cell stemness [21]. Recent publication showed reciprocal interactions between CAFs and macrophages. CAFs polarizes macrophages to a pro-angiogenic M2 phenotype while macrophages enhances CAF induced tumor invasiveness [22]. However, we did not observe any contribution from CAFs during TAM stimulation of sphere formation in our study. Thus we speculate that the interaction between CAFs and macrophages may regulate tumor invasion, but the regulation of tumor stemness and tumor initiation by these two cells types may be independent events. We have shown that even though macrophages from SMAD3KO and LCArg1 mice produce normal levels of TGFβ they were unable to stimulate sphere formation. This again supports the idea that synergism with polyamines is necessary and that CAF derived TGFβ alone will similarly be unable to stimulate TICs.

In conclusion, our results demonstrate that TAMs interact specifically with the CD34^−^ TIC population from RETAAD tumors, both *in vitro* and *in vivo*. We show that macrophage-derived TGFβ and polyamines are essential for TIC survival and can support their resistance to chemotherapeutic drugs. We also show that changes in the macrophage-responsiveness of TICs mirror tumor progression *in vivo* and that macrophages may accelerate cancer progression in mice with slow rates of tumor development. *In vivo* macrophage inhibition transiently suppresses the macrophage-responsiveness of the CD34^−^ TIC population, whereas chemotherapy accelerates the emergence of TAM-responsive TICs. The identification and further characterization of this macrophage-responsive TIC population will facilitate development of improved therapies for human cancers.

## MATERIAL AND METHODS

### Mice

Animal care and experimental procedures were approved by the Singapore IACUC under protocols 120742 and ARF-SBS/NIE-A0167-AZ, or performed under Animal Study Proposal LPD16E approved by the NIAID IACUC. RETAAD mice were generated as described [55]. Male and female mice 23-60 weeks of age were used for experiments. Smad3KO mice [56], *Arg1^fl/fl^; LysMcre* mice and *Arg1^fl/fl^; Tie2cre* mice [34] were generated as described.

### Cell culture

Tumors were dissociated with collagenase A (1mg/ml) and DNase I (0.1mg/ml; Roche) and cultured in stem cell medium [DMEM/F12 (1:1), 1% penicillin/streptomycin, B27 supplement (Invitrogen), 10ng/ml bFGF and 20ng/ml EGF (Peprotec)] at 4000 cells/cm^2^ in ultra-low attachment culture wells (Corning). Culture plates were not disturbed during the culturing period to avoid sphere formation by aggregation. Spheres greater than 30μm in diameter were counted 5-7 days after cell seeding. Immune cells were added at a ratio of 1:50 to tumor cells and remained for the duration of the culture.

### Cell selection

TIC and immune cell populations were harvested from the same mice for each experiment. CD45^+^ immune cells were depleted from the tumor with anti-CD45-PE antibody and the anti-PE EasySep selection kit (StemCell). Tumor cell sub-populations were sorted by flow cytometry using CD34-biotin (eBioscience), anti-CD271 (Millipore) and CD45-FITC (Biolegend) antibodies followed by streptavidin-PE and anti-rabbit PECy7 (Invitrogen) antibodies. Immune cell population were sorted using CD45, CD3, CD4, CD8, NK1.1, CD19, CD11b (Biolegend), and CD68 (AbDserotec) conjugated to appropriate fluorophores. Stromal cell populations were sorted using CD31-PECy7 and PDGFRα-APC (Biolegend).

### *In vivo* CD115 inhibition

Mice underwent gavage with Ki20227 (30mg/kg/day in 0.05% methylcellulose; Accelachembio) over 10 days. The anti-CD115 monoclonal antibody [clone AFS98; [57]] was purified from culture supernatant of AFS98 hybridoma. 3mg of antibody was injected intra-peritoneally on days 0, 1 and 7. Mice were sacrificed on day 10.

### Transplantation of spheres into mice

Melanospheres (100 per mouse) were manually picked under the microscope and re-suspended in 100μl of PBS for retro-orbital injection into Rag1 mice. Mice were sacrificed after 8 weeks and the lungs harvested for immunohistochemistry.

### BMDM and conditioned medium

Bone marrow cells were cultured in complete medium [DMEM, 10% FBS, 1% penicillin/streptomycin, 1% L-glutamine, 30% L929 cell-conditioned medium] for 5 days and polarized towards M1 (100ng/ml LPS; Sigma, and 20ng/ml IFN-γ; Miltenyi) or M2 (20ng/ml IL-4; Miltenyi) phenotypes with cytokines overnight. BMDM-conditioned medium (CM) was generated by replacing complete medium with stem cell medium and harvesting after 24 hours and used at 1/10^th^ of stem cell medium. Inhibitors were added during BMDM polarization and cells were washed thoroughly prior to CM generation.

### Inhibitors and stimulators

All reagents were added at the start of the sphere culture and remained for the duration of the experiment. Ki20227, SD208 and DFMO (Tocris), L-norvaline, putrescine, spermine and spermidine (Sigma), TGFβ (R&D systems), anti-TGFβ (clone 1D11, R&D Systems) and anti-CD115 antibodies were added at the concentrations stated on the respective figures. For pretreatment experiments, cells were incubated with anti-CD115 (1μg/ml) for 1 hour then washed thoroughly prior to culturing. Temozolamide and cisplatin (Sigma) were used at 200μg/ml and 0.5ug/ml respectively *in vitro*. For *in vivo* chemotherapy, temozolamide (3mg) was injected intra-peritoneally on days 0, 1 and 2. Mice were sacrificed on day 7.

### Statistical analysis

Graphs and statistical analysis were generated using Graphpad Prism 6 software. Tests applied are indicated in the figure legends. Two-tailed non-parametric tests (Mann-Whitney for unpaired and Wilcoxon matched-pair test for paired experiments) were used for comparison between two groups. One way ANOVA was used to compare multiple groups with one experimental parameter while a two-way ANOVA was used to compare multiple groups with two experimental parameters.

Additional experimental procedures can be found in the Supplemental section.

## SUPPLEMENTARY MATERIAL AND FIGURES


