# Supplementary material for: Melanoma-initiating cells exploit M2 macrophage TGFβ and arginase pathway for survival and proliferation
Source: Oncotarget. 2014 Sep 16;5(23):12027–42. doi: 10.18632/oncotarget.2482 (PMC4322977; doi:10.18632/oncotarget.2482)
Supplement: Supplementary file 1 [file oncotarget-05-12027-s001.pdf]

Melanoma-initiating cells exploit M2 macrophage TGFβ and arginase pathway for survival and proliferation

Supplementary Material

Immunohistochemistry. Formalin-fixed paraffin-embedded sections (5µm) were immunolabeled for S100B to identify melanoma cells and Ki67 to identify proliferating cells. Briefly, sections were dewaxed in xylene and heat-treated in Target Retrieval Solution (DAKO). Sections were blocked with 3% (v/v) hydrogen peroxide, biotin and avidin block (DAKO) and 10% (v/v) normal goat serum (DAKO). Rabbit anti-S100B (DAKO; 1:4,000) and rat anti-Ki67 (DAKO; 1:40) antibodies were applied and incubated overnight at 4°C. S100B was revealed with anti-rabbit HRP (DAKO) and AEC peroxidase substrate (Vector Laboratories). Ki67 was revealed with biotinylated donkey anti-rat (Jackson Lab; 1:300) followed by alkaline phosphatase-conjugated strepavidin (Rockland Inc.; 1:2000) and Alkaline Phosphate Substrate Kit III (Vector Laboratories). OCT frozen sections were immunolabeled for S100B, CD45 and CD68. Sections were rehydrated, blocked with biotin and avidin block (DAKO) and 10% (v/v) normal goat serum (DAKO). Anti-S100B (DAKO), CD45-APC (Biolegend; 1:100) and CD68-biotin (AbDserotec; 1:100) antibodies were applied and incubated overnight at 4°C. Anti-rabbit-FITC (DAKO; 1:500) and Strepavidin-PE (Invitrogen; 1:500) antibodies were used to reveal S100B and CD68 respectively. Sorted cells were cytospun onto slides and stained with 0.3% May-Grunwald stain followed by 8% Geimsa stain.

Microarray. $CD34^-$, $CD34^-$+M2-CM and $CD34^+$ spheres were manually harvested under the microscope. RNA was extracted with ABI Arcturus Picopure RNA isolation kit and labeled with Epicentre TargetAmp™ 2-Round Biotin-aRNA Amplification Kit 3.0. Hybridization was performed on the Illumina Mouse WG-6 v3 beadchip. The chip was scanned with BeadArray Scanner 500GX and analyzed with GenomeStudio Gene Expression v 1.9.0. Principal component analysis was performed in R v 2.15.2 and Bioconductor. The co-ordinates for the first 3 principal components were plotted with the 3D visualization option in Tibco Spotfire (http://spotfire.tibco.com).

TGFβ and Arginase assays. TGFβ concentration in the CM was measured using the TGFβ1 Quantikine Elisa kit (R&D Systems) as described by the manufacturer. Arginase activity assay was performed as described in [58].

Gene expression analysis. Cells were homogenized in TRIZOL (Qiagen). RNA was extracted using the Qiagen RNeasy Micro kit. cDNA was reverse transcribed (Roche Applied Biosystem reagents) and subjected to quantitative PCR with SYBR green (Bio-Rad) and specific primers. CD34 (5'-CCACCGAGCCATATGCTTAC-3' and 5'-AGCAGGGAGCAGACACTAGC-3'), CD115 (5'- TCATGAGTCACCTGGGACAG-3' and 5'- CCCTGCGCACATATTTCTTC-3'), NOS2 (5'-CAAACCCAAGGTCTACGTTCAG-3' and 5'-CCACCAGCTTCTTCAATGTG-3'),

IL-1β (5'- AAAGCTCTCCACCTCAATGG-3' and 5'- TCTTCTTTGGGTATTGCTTGG-3'), IL-12p40 (5'-CAAATTACTCCGGACGGTTC-3' and 5'-AGTCCCTTTGGTCCAGTGTG-3'), TGFβ (5'-GGCTACCATGCCAACTTCTG-3' and 5'-GCTTGCGACCCACGTAGTAG-3'), Arginase1 (5'-CAAGACAGGGCTCCTTTCAG-3' and 5'-GTAGTCAGTCCCTGGCTTATGG-3') and FIZZ1 (5'-CCTGCTGGGATGACTGCTAC-3' and 5'-CGAGTAAGCACAGGCAGTTG-3'). Gene expression was normalized to GAPDH (5'-TGCGACTTCAACAGCAACTC-3' and 5'-ATGTAGGCCATGAGGTCCAC-3').

Supplemental Figure S1

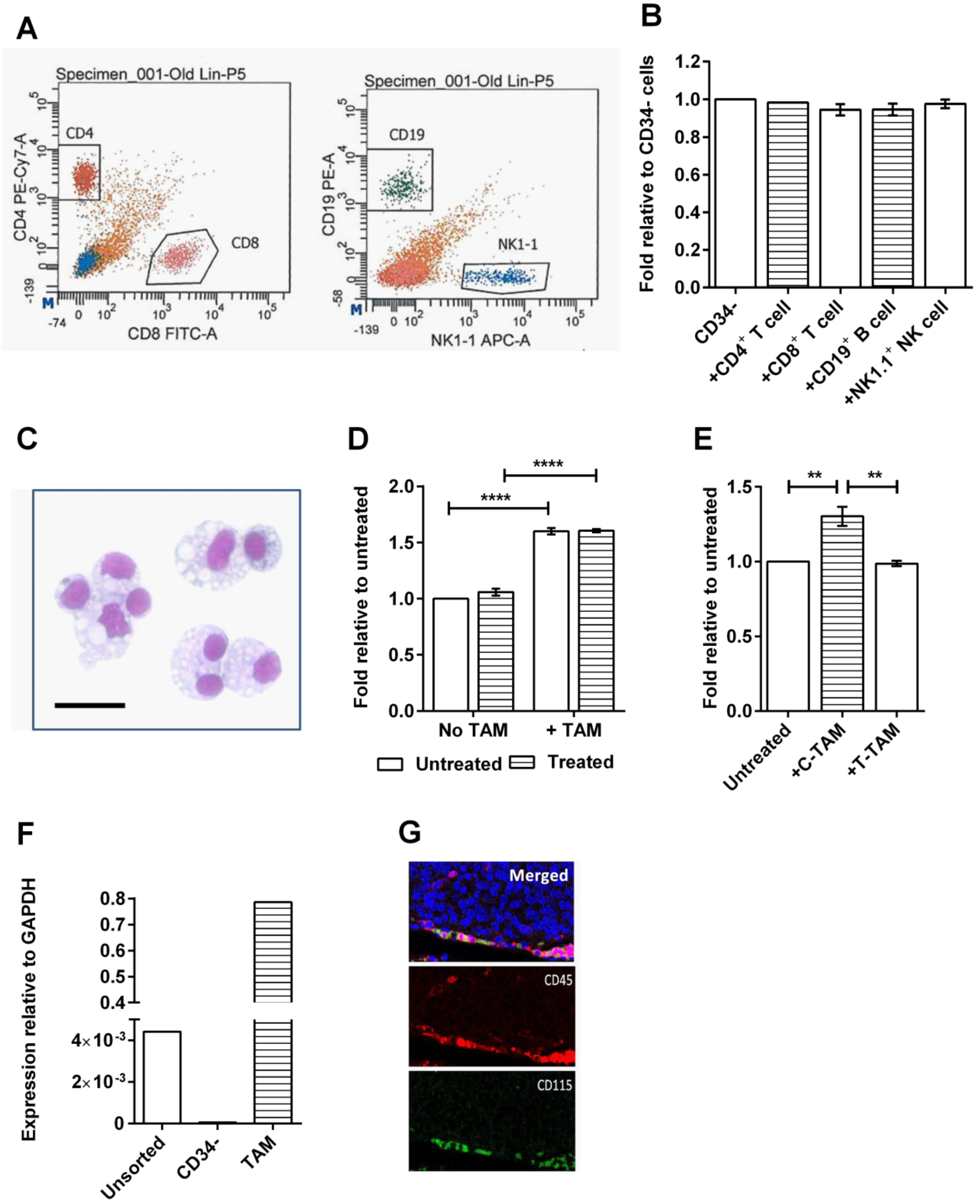

Supplemental Figure S1: Characteristics of tumor-derived immune cells.

(A) $CD45^+$ cells separated into $CD4^+$ T cells, $CD8^+$ T cells, $CD19^+$ B cells and $NK1.1^+$ NK cells by flow cytometry.

(B) The immune populations isolated in panel **(A)** did not stimulate melanosphere formation from $CD34^-$ TICs.

(C) Brightfield image of $CD11b^+CD68^+$ TAMs showing macrophage morphology. Cells were cytospun onto slides and stained with May-Giemsa stain. Scale bar equals 20μm.

(D) Pretreatment of tumor cells: tumor cells were untreated or pre-treated with anti-CD115 prior to culturing with (+TAM) or without (No TAM) TAMs. Bars represent mean ± SE, **** $P<0.0001$, 2-way ANOVA.

(E) Pretreatment of macrophages: tumor cells were cultured with untreated TAMs (C-TAM) or TAMs pre-treated with anti-CD115 (T-TAM) before adding to tumor cells. Bars represent mean ± SE, ** $P<0.01$, 1-way ANOVA

(F) Representative graph comparing CD115 gene expression in unsorted tumor cells, CD34- tumor cells and TAMs.

(G) Immunofluorescent image of tumor showing CD115 expression (green) on $CD45^+$ cells (red). Nuclei are in blue.

Supplemental Figure S2

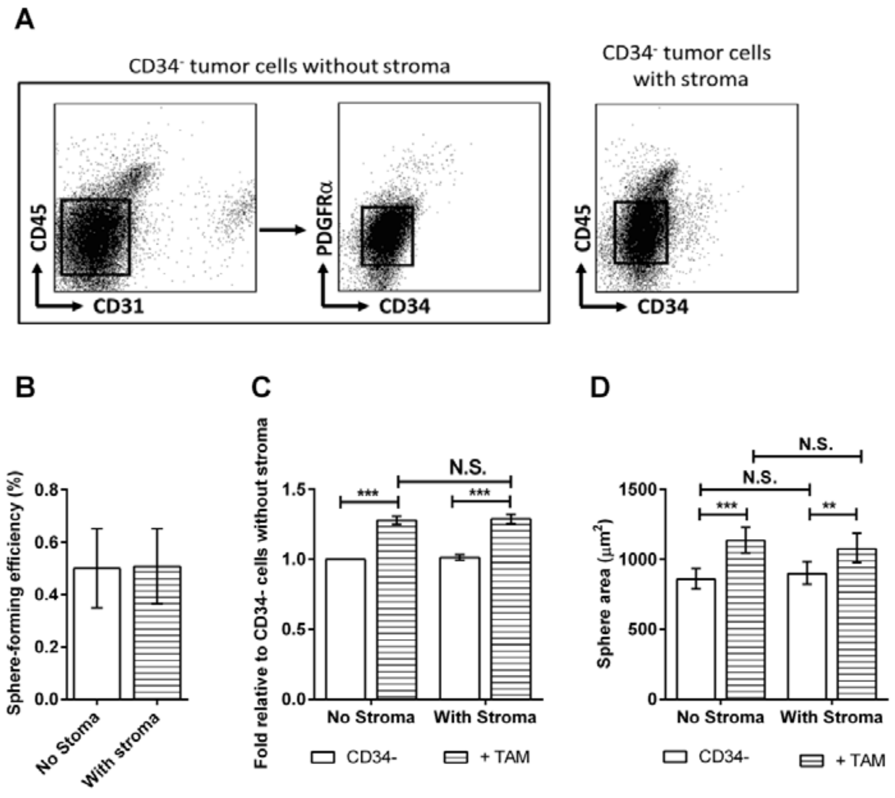

Supplemental Figure S2: Stomal cells do not affect TAM stimulated sphere formation.

(A) Flow cytometry plot showing the selection for $CD34^-$ tumor cells with and without stromal cells. Stromal cells were identified as negative for CD45 and positive for PDGFRα and CD31. Antibodies for PDGFRα and CD31 were not included when the stromal cell population was not excluded.

(B) Graph showing the sphere formation efficiency from $CD34^-$ cells sorted in panel (**A)** with and without stromal cells. Stromal cells do not affect sphere formation efficiency of $CD34^-$ tumor cells. Bars represent mean ± SD.

(C) Graph comparing the stimulatory effect of TAMs on sphere formation from $CD34^-$ tumor cells in the presence and absence of stromal cells. Stromal cells do not affect the stimulatory activity of TAMs. Bar graphs are expressed as fold change in sphere-forming efficiency relative to untreated $CD34^-$ TICs without stromal cells. Bars represent mean ± SE, ***$P < 0.001$, N.S. not significant, 2-way ANOVA.

(D) Graph comparing the stimulatory effect of TAMs on the size of spheres formed from $CD34^-$ tumor cells in the presence and absence of stromal cells. Stromal cells do not affect the size of spheres or the stimulatory effect of TAMs. Bars represent geometric mean ± 95% CI, **$P < 0.01$, ***$P < 0.001$, N.S. not significant, 2-way ANOVA.

Supplemental Figure S3

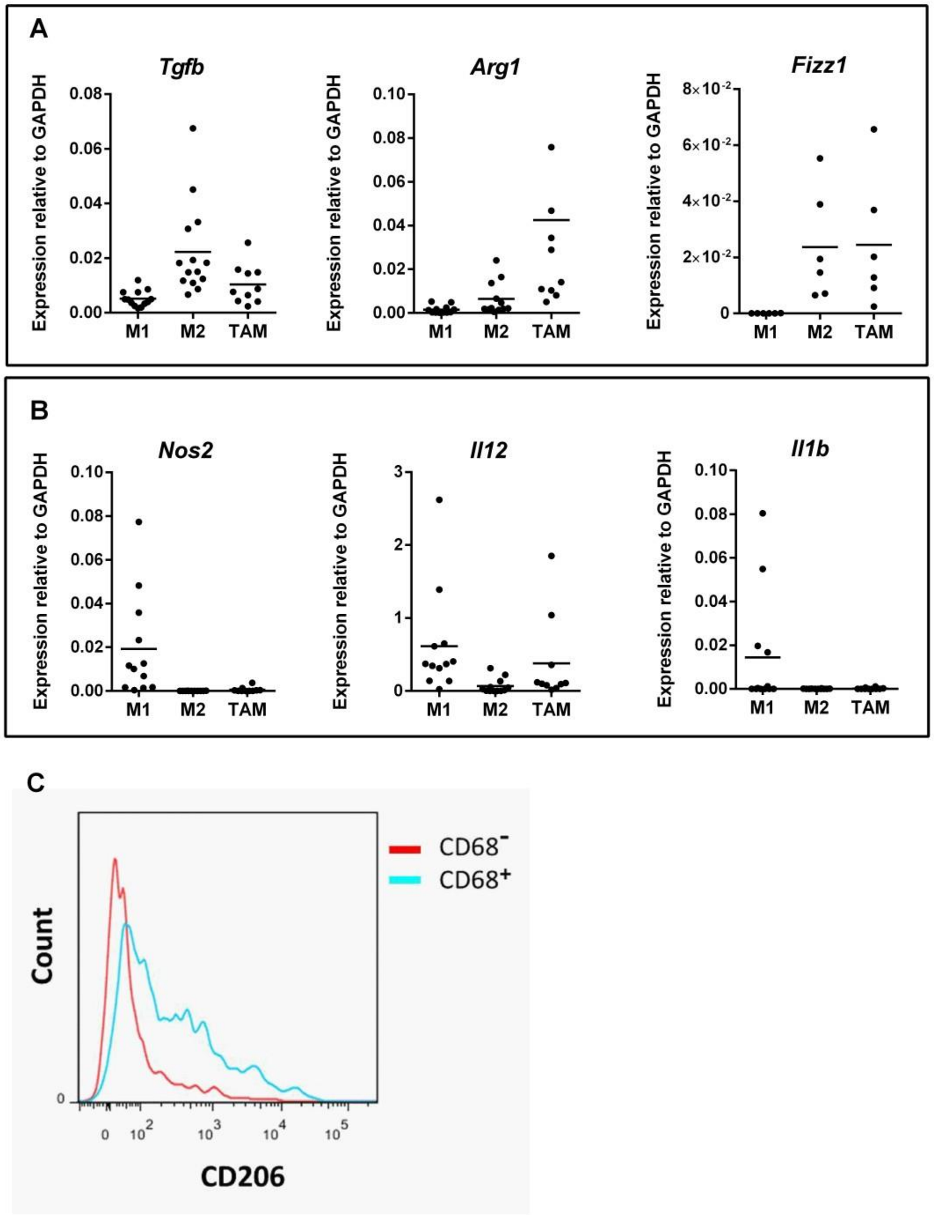

Supplemental Figure S3: Gene analysis of tumor-derived macrophages.

(A) Graph comparing expression of M2-related genes in M1- and M2- polarized BMDM and TAMs. Bars represent mean with each point representing cells from one mouse.

(B) Graph comparing expression of M1-related genes in M1- and M2-polarized BMDM and TAMs. Bars represent mean with each point representing cells from one mouse.

(C) Flow cytometry histogram comparing CD206 staining on CD11b$^+$CD68$^+$ and CD11b$^+$CD68$^-$ cells.

Supplemental Figure S4

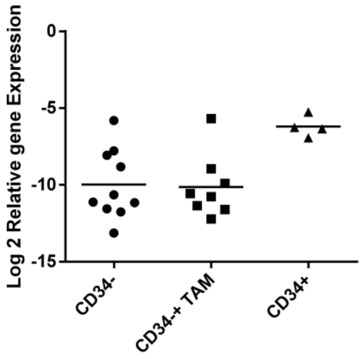

Supplemental Figure S4: Tumor-associate macrophage do not alter CD34 expression.

Graph showing the Log2 gene expression of CD34 relative to GAPDH in spheres cultured from $CD34^-$ cells, $CD34^-$ cells stimulated with TAMs and $CD34^+$ cells. Bars represent mean with each point representing cells from one mouse.

Supplemental Figure S5

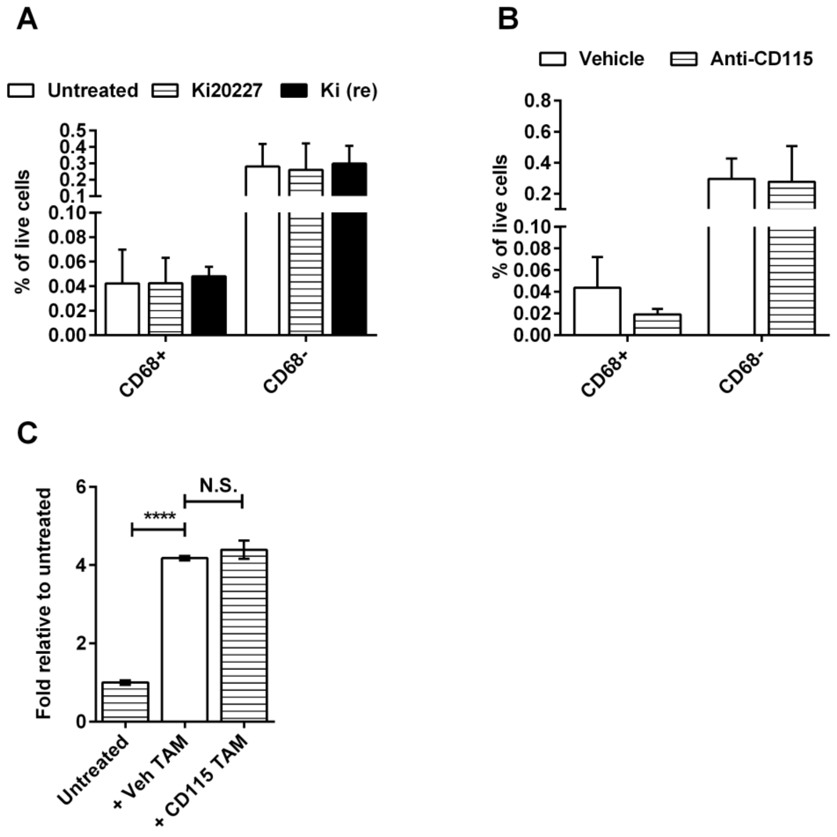

Supplemental Figure S5: Effects of macrophage depletion *in vivo*.

(**A**) Graph showing effect of Ki20227 treatment and recovery from Ki20227 treatment on the percentage of CD68$^+$ cells within the tumor. Bars represent mean ± SD.

(**B**) Graph showing effect of anti-CD115 treatment on the percentage of CD68$^+$ cells within the tumor. Bars represent mean ± SD.

(**C**) Graph showing the stimulatory property of TAMs derived from vehicle- and anti-CD115- treated mice. CD34$^-$ cells from untreated mice were cultured with macrophages from vehicle- (Veh TAM) or anti-CD115-treated (CD115 TAM) mice. Bars represent mean ± SE, **** P<0.0001, 1-way ANOVA N.S.: not significant.

Supplemental Figure S6

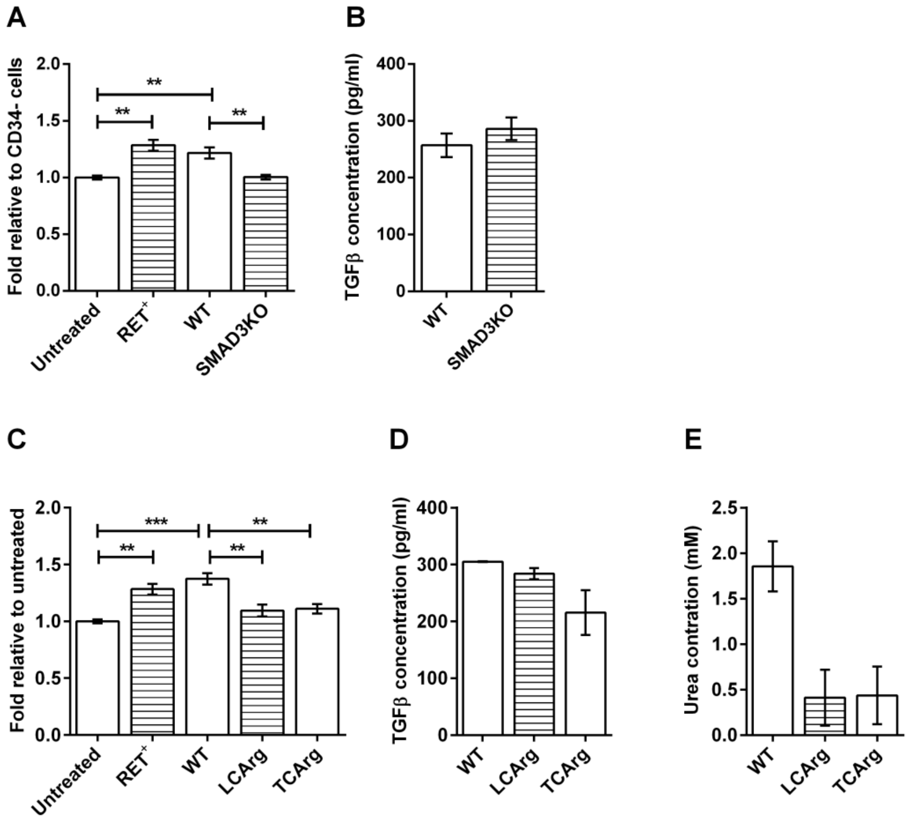

Supplemental Figure S6: Macrophages stimulate melanosphere formation via TGFβ and arginase pathway.

(A) Graph showing the effect of CM derived from M2-BMDM from tumor bearing mice (RET^+), wild- type mice (WT) or Smad3 knockout mice (Smad3KO) on melanosphere formation. Bars represent mean ± SE, **$P<0.01$, 1-way ANOVA.

(B) Representative graph comparing the TGFβ level within the CM derived from M2-BMDM from WT or SMAD3KO mice. Bars represent mean ± SD.

(C) Graph showing the effect of CM derived from M2-BMDM from tumor bearing mice (RET^+), *$Arg1^{fl/fl}$* (WT), *$Arg1^{fl/fl}$;LysMcre* (LCArg) and *$Arg1^{fl/fl}$;Tie2cre* (TCArg) mice on melanosphere formation. Bars represent mean ± SE, ** $P<0.01$, *** $P<0.001$, 1-way ANOVA.

(D) Representative graph comparing the TGFβ level within the CM derived from M2-BMDM from WT, LCArg and TCArg mice. Bars represent mean ± SD.

(E) Representative graph showing Arginase activity in M2- polarized WT, LCArg and TCArg BMDM. Bars represent mean ± SD.

Supplemental Figure S7

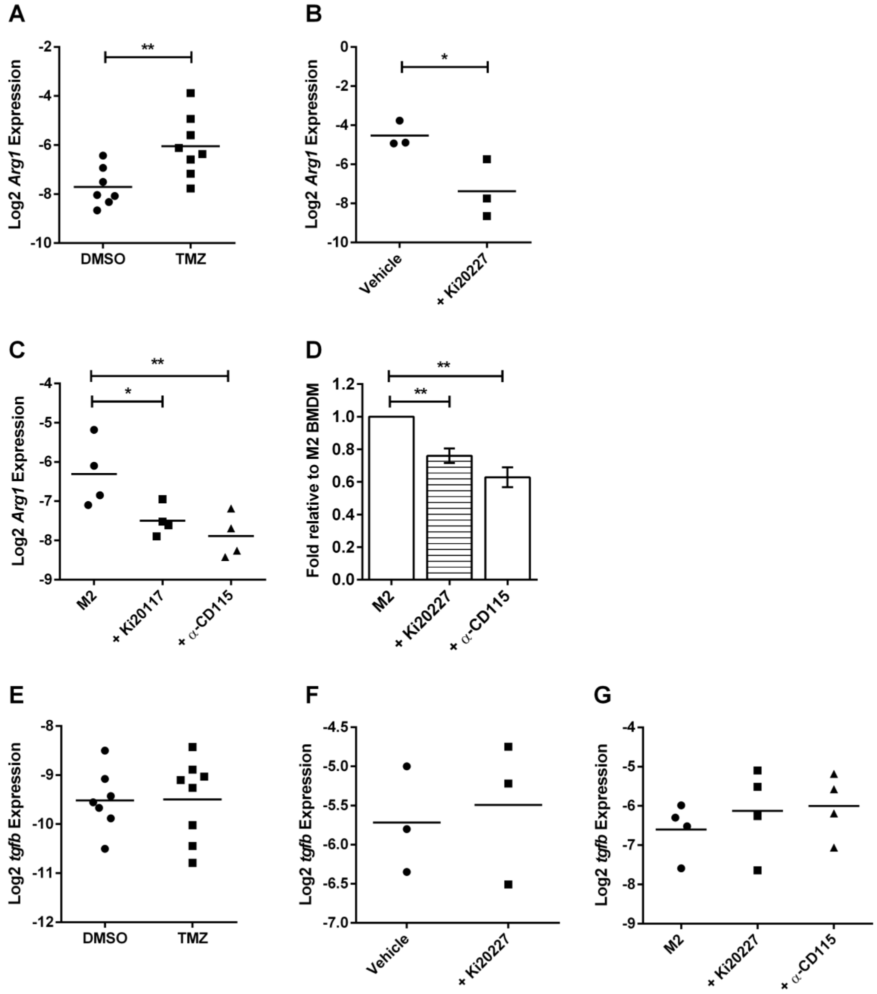

Supplemental Figure S7: The stimulatory effect of tumor-associated macrophages depend on Arginase

(A) In vivo treatment with TMZ increased gene expression of Arg1 in TAMs. Graph showing the Log2 gene expression of *Arg1* relative to GAPDH from TAMs isolated from DMSO or TMZ treated mice. Bars represent mean with each point representing cells from one mouse, ** $P<0.01$, unpaired t test.

(B) In vivo inhibition of CD115 decreased gene expression of Arg1 in TAMs. Graph showing the Log2 gene expression of *Arg1* relative to GAPDH from TAMs isolated from vehicle or Ki20227 treated mice. Bars represent mean with each point representing cells from one mouse, * $P<0.05$, unpaired t test.

(C) In vitro inhibition of CD115 decreased gene expression of Arg1 in M2-BMDM. Graph showing the Log2 gene expression of Arg1 relative to GAPDH from M2-BMDM treated with Ki20227 or anti- CD115. Bars represent mean with each point representing cells from one mouse, * $P<0.05$, ** $P<0.01$, 1-way ANOVA.

(D) In vitro inhibition of CD115 reduced Arg1 activity. Graph showing fold change relative to untreated M2-BMDM in urea concentration as a measure of Arg1 activity. Bars represent mean ± SE, ** $P<0.01$, 1-way ANOVA.

(E) In vivo treatment with TMZ did not affect *tgfb* gene expression in TAMs. Graph showing the Log2 gene expression of *tgfb* relative to GAPDH from TAMs isolated from DMSO or TMZ treated mice. Bars represent mean with each point representing cells from one mouse.

(F) In vivo inhibition of CD115 did not affect *tgfb* expression in TAMs. Graph showing the Log2 gene expression of *tgfb* relative to GAPDH from TAMs isolated from vehicle or Ki20227 treated mice. Bars represent mean with each point representing cells from one mouse.

(G) In vitro inhibition of CD115 did not affect *tgfb* expression in M2-BMDM. Graph showing the Log2 gene expression of *tgfb* relative to GAPDH from M2-BMDM treated with Ki20227 or anti-CD115. Bars represent mean with each point representing cells from one mouse.
